# Sarcoid-like lesions obfuscating the diagnosis of disseminated *Mycobacterium genavense* infection in a patient with IL-12Rβ1-associated immunodeficiency

**DOI:** 10.1186/s12879-022-07644-4

**Published:** 2022-10-04

**Authors:** Sara Denicolò, Sophie Laydevant, Julia Fink, Christoph Geiger, Alex Pizzini, Mario Sarcletti, Johannes Zschocke, Rosa Bellmann-Weiler, Günter Weiss, Ivan Tancevski

**Affiliations:** 1 Department of Internal Medicine II (Infectious Diseases, Pneumology and Rheumatology), Medical University Innsbruck, Anichstr. 35, 6020 Innsbruck, Austria; 2 Department of Internal Medicine IV (Nephrology and Hypertensiology), Medical University Innsbruck, 6020 Innsbruck, Austria; 3 Department of Dermatology, Venereology and Allergology, Medical University Innsbruck, 6020 Innsbruck, Austria; 4 Institute of Human Genetics, Medical University Innsbruck, 6020 Innsbruck, Austria

**Keywords:** *Mycobacterium genavense*, *IL-12Rβ1* deficiency, Sarcoid-like reaction, Interferonopathy, Case report

## Abstract

**Background:**

Sarcoidosis is a systemic inflammatory disease that is characterized by non-caseating epithelioid-cell granulomas upon histology. However, similar histological findings may also be seen with certain infections. Thus, differentiation from infection is pivotal to ensure appropriate treatment. Here, we present a case of a disseminated infection with *Mycobacterium genavense* owing to an interleukin 12 receptor subunit beta 1 (IL-12Rβ1) associated immunodeficiency in a previously healthy female who was initially misdiagnosed with sarcoidosis. *M. genavense* is a nontuberculous mycobacterium which can cause lymphadenopathy, gastrointestinal and bone marrow infiltration in immunocompromised patients. With this case report we aim to highlight that an infection with *M. genavense* on the ground of a genetic defect of mycobacterial immune control may represent a rare differential diagnosis of sarcoidosis.

**Case presentation:**

A 31-year-old female was referred to our hospital with progressive lymphadenopathy, hepatosplenomegaly, pancytopenia and systemic inflammation. She had previously been evaluated for generalized lymphadenopathy in another hospital. At that time, lymph node biopsies had revealed sarcoid-like lesions and a systemic corticosteroid treatment was initiated based on a putative diagnosis of sarcoidosis. When her condition worsened, she was transferred to our university clinic, where the diagnosis of disseminated *M. genavense* infection owing to an inborn interferonopathy was made. Her family history revealed that her brother had also suffered from IL-12Rβ1 deficiency and had died from a systemic infection with *M. genavense* at the age of 21. The patient received antimycobacterial treatment combined with subcutaneous type I interferon, which eventually led to a gradual improvement over the next months.

**Conclusions:**

Differentiating between sarcoidosis and sarcoid-like lesions secondary to infections may be challenging, especially when pathogens are difficult to detect or not expected in an apparently immunocompetent patient. Patients with IL-12Rβ1*-*associated immunodeficiency may be asymptomatic until adulthood, and disseminated *M. genavense* infection on the grounds of an IL-12Rβ1*-*associated immunodeficiency may represent a rare differential diagnosis of sarcoidosis.

## Background

Sarcoidosis is an inflammatory multisystemic disease, and the diagnosis is established when clinical and radiological findings are compatible with the disease, and non-caseating epithelioid-cell granulomas are histologically described [1, 2]. However, the latter must be differentiated from sarcoid-like lesions secondary to infections, drugs, malignancy or other causes [1, 2]. Differentiation from infection can be challenging, especially when pathogens are rare and difficult to detect or not expected in an apparently immunocompetent patient.

Here, we present a case of a disseminated infection with the opportunistic pathogen *Mycobacterium genavense* owing to an interleukin 12 receptor subunit beta 1 (IL-12Rβ1) associated immunodeficiency in a previously healthy female who was initially misdiagnosed with sarcoidosis.

IL-12Rβ1 deficiency is an autosomal recessive, hereditary immunodeficiency that is part of a complex of genetic disorders termed Mendelian susceptibility to mycobacterial diseases (MSMD) [3]. MSMD comprises a number of rare genetic defects that affect the interaction between macrophages and T-cells and interfere with IFN-γ production [3]. Affected patients are susceptible to recurrent and disseminated infections with mycobacteria and other intracellular pathogens [3]. IL-12Rβ1 deficiency is the most common defect, and to date, mutations in 18 different genes associated with MSMD have been documented [3–5]. Patients with immunodeficiency caused by mutations in the IL-12Rβ1 gene often become symptomatic with infections in childhood. However, they may also remain asymptomatic until adulthood as the clinical phenotype is highly variable [3, 5].

Disseminated infections with *M. genavense* were first described in patients with human immunodeficiency virus (HIV), but have also been reported in non-HIV immunocompromised patients [6, 7]. Mahmood et al [7]. summarized 46 cases of *M. genavense* infections in non-HIV immunocompromised hosts, and found that 14% were associated with immunosuppressive treatment for sarcoidosis. With this case report we aim to highlight that an infection with *M. genavense* on the ground of a genetic defect of mycobacterial immune control may represent a rare but important differential diagnosis of sarcoidosis.

## Case presentation

A 31-year-old female was referred to our tertiary care hospital with progressive pancytopenia, hepatosplenomegaly and systemic inflammation.

Twelve months prior to admission, she had been evaluated for right lower quadrant abdominal pain in another hospital. At that time, the diagnostic workup revealed terminal ileitis and a generalized lymphadenopathy. The biopsy of a mesenteric and a retroperitoneal lymph node showed granulomatous / sarcoid-like lesions upon histological evaluation. Based on the suspected diagnosis of sarcoidosis, a therapy with 0.5 mg Prednisolone per kilogram of body weight was initiated.

**Fig. 1:**
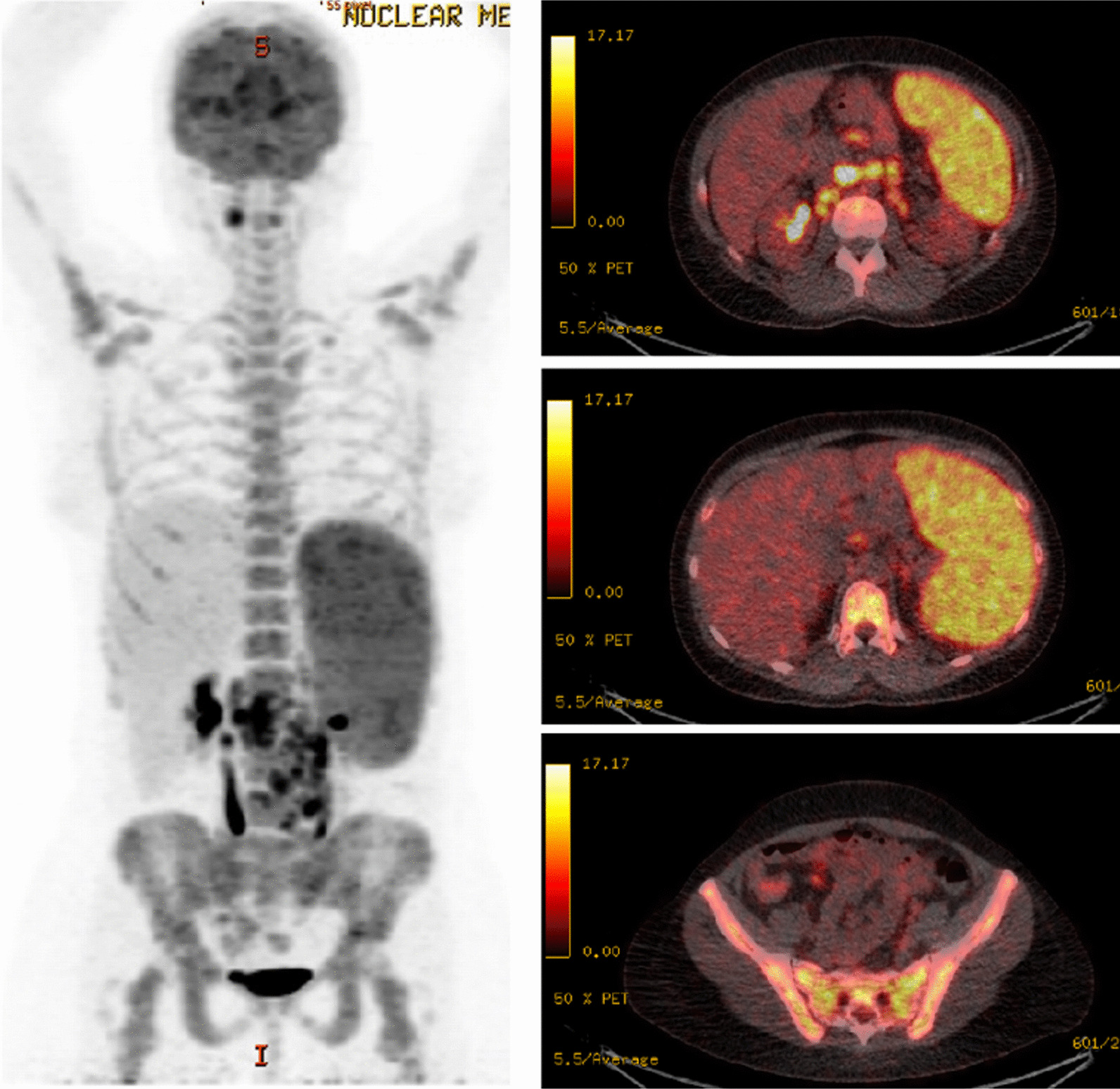
^18^FDG-PET analysis shows hypermetabolic lymphadenopathy and splenomegaly, together with enhanced tracer uptake in the small intestine, as well as a hypermetabolic bone marrow

Over the following months, the patient’s condition gradually worsened, and she was transferred to our hospital for further evaluation. On admission, she complained of recurrent fever, night sweats and abdominal pain with predominance in the upper left quadrant. Upon physical examination, the patient was found to have sinus tachycardia and left upper abdominal quadrant tenderness. Blood pressure and oxygen saturation were within normal limits. Laboratory results showed new pancytopenia (leukocytes 1.8 G/l [reference range 4.0–10.0 G/l], hemoglobin 8.5 g/dl [reference range 12.0–15.7 g/dl], thrombocytes 72 G/l [reference range 150–380 G/l]) and systemic inflammation with a C-reactive protein of 14 mg/dl (reference range < 0.5 mg/dl), and an elevated erythrocyte sedimentation rate of 107 mm/h (reference range < 20 mm/h). Renal function, liver enzymes, and lactate dehydrogenase were within normal ranges, chest X ray and urine analysis showed no appreciable disease. ^18^FDG-PET/CT revealed progressive generalized and hypermetabolic lymphadenopathy (especially of mesenteric and retroperitoneal lymph nodes), a pronounced hypermetabolic splenomegaly, enhanced tracer uptake in the small intestine, and diffuse bone marrow hypermetabolism (Fig. 1). Serological or molecular analyses for *human immunodeficiency virus, Epstein–Barr virus, cytomegalovirus, human herpesvirus 8*, *Salmonella typhi, Leishmania spp., Francisella tularensis* and *Yersinia enterocolitica* were negative. Autoimmune testing, including serum ACE and sIL2R, was negative.

The patient was of Turkish descent, and was born and raised in Germany. She had no premedication or allergies at the time of first presentation, and had not suffered from any serious acute or recurrent infections in the past. Importantly, examination of the patient’s family history revealed that her brother had died from a systemic infection with *M. genavense* at the age of 21, with an underlying IL-12Rβ1 deficiency. Her parents were both healthy and she had no other siblings. Neither the parents nor the patient had been genetically tested until the time of admission to our hospital.

**Fig. 2:**
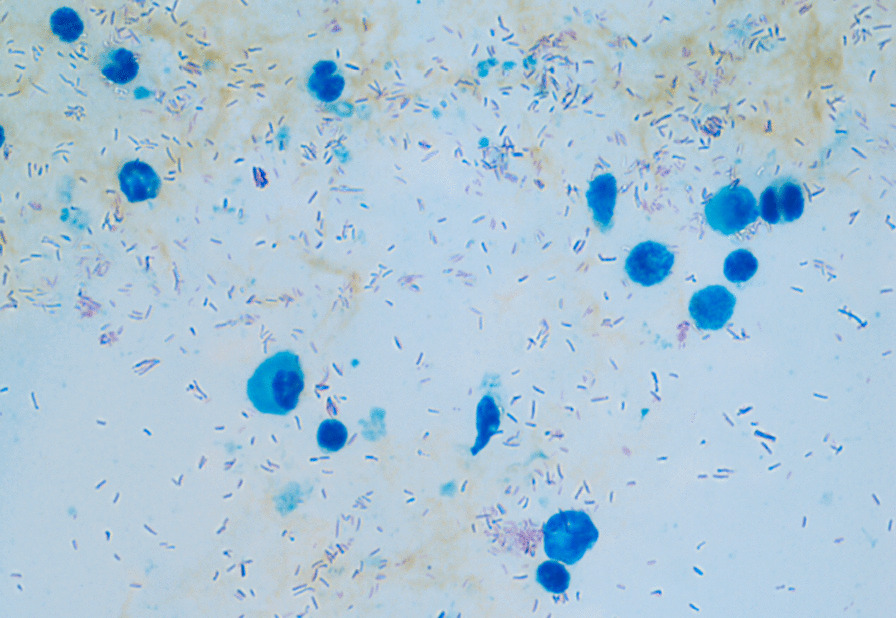
Ziehl–Neelsen staining of a bone marrow sample with numerous acid-fast bacilli and a dramatically reduced hematopoiesis

At the time of the patient`s first presentation one year prior to the diagnosis of an IL-12Rβ1 deficiency, biopsy specimen of terminal ileum and lymph nodes were sent to two independent pathology laboratories. Ziehl–Neelsen staining was negative in all specimen. In one laboratory real-time PCR for Mycobacterium spp. *(M. tuberculosis, M. bovis, M. bovis BCG, M. africanum, M. microti, M. canetti, M. pinipedii;* Amplisens^®^ MTC-FRT PCR Kit) was negative, while an independent laboratory reported a positive PCR result for atypical mycobacteria (LCD-Array Kit, Chipron). However, it did not provide further species information, as the additional testing of a predefined panel of nontuberculous mycobacteria (*M. avium complex, M. kansasii, M. xenopi, M. abscessus, M. gordonae, M. peregrinum, M. szulgai, M. haemophilus, M. marinum/ulcerans, M. simiae and M. smegmatis)* was negative*.* In our workup, Ziehl–Neelsen staining of bone marrow revealed massive infiltration with acid-fast bacilli (Fig. 2), and mycobacterial histiocytosis with displacement of hematopoietic cells was described. Pan-bacterial PCR of a bone marrow sample unveiled *M. genavense*, which was later confirmed by mycobacterial culture. Attempts to culture the pathogen from blood samples were not successful. With the diagnosis of disseminated infection with *M. genavense* and the positive family history, genetic investigations were initiated. These identified a homozygous pathogenic variant p(Cys198Arg) (c.592T > C) in the *IL12RB1* gene (NM_005535, rs121434495), previously reported in attenuated IL-12Rβ1-associated immunodeficiency [8].

An antimycobacterial treatment with rifampin 600 mg q.d., ethambutol 1600 mg q.d. and clarithromycin 500 mg b.i.d. was initiated. After three weeks of treatment the patient developed erythema multiforme, and antimycobacterial treatment was switched to azithromycin 500 mg q.d., moxifloxacin 400 mg q.d., rifabutin 300 mg q.d., and intravenous amikacin 1000 mg q.d. The antimycobacterial treatment was combined with subcutaneous IFN-γ at 50 µg/kg body weight three times a week. The patient improved gradually over the next months. Amikacin was administered for 24 weeks and then discontinued due to tinnitus without hearing impairment upon tone audiogram. The treatment consisting of azithromycin, moxifloxacin, rifabutin and IFN-γ is ongoing at the time of submission.

## Discussion and conclusions

*M. genavense* is a rare environmental mycobacterium that may cause opportunistic infections in immunocompromised patients, and it was first described in patients with HIV in the early 1990s [6]. Patients typically present with fever, night sweats, weight loss, hepatosplenomegaly, (especially mesenteric) lymphadenopathy, gastrointestinal symptoms, and high immune activation [7]. *M. genavense* is difficult to detect as it needs special conditions for cultivation and does not grow in standard mycobacterial media [6]. Although PCR has a high sensitivity, *M. genavense* is usually not part of PCR panels that are used for the detection of tuberculous and nontuberculous mycobacteria in routine clinical settings. Ziehl–Neelsen staining, on the other hand has a significantly lower sensitivity because of a detection limit of approximately 5,000–10,000 bacteria per ml [9]. In addition, *M. genavense* is a slow growing pathogen with low bacterial concentration, thus Ziehl–Neelsen staining may be false negative. Interferon-γ Release Assays are not adequate for the detection of nontuberculous mycobacteria and are not reliable in patients with IL-12Rβ1 deficiency, as an intact IL12/IFN-γ axis is needed [10]. In the here presented case, the diagnosis was established by bone marrow aspiration and subsequent pan-bacterial PCR.

*M. genavense* has been suggested to have a ubiquitous distribution as it was identified in tap water, the intestinal tract of immunocompetent humans and animals [11–14]. An intestinal/faecal-oral transmission has been theorized [15], however no human-to-human transmission has been reported so far. As the patient`s brother died of the same mycobacterial infection, infection from the same environmental exposure seems most likely, although a direct transmission cannot be fully excluded. The time of infection could not be determined.

Mycobacterial antigens have been discussed as trigger for sarcoidosis [16]. However, sarcoidosis secondary to an exposure to *M. genavense* seems unlikely in this case, as no improvement was observed upon previous corticosteroid therapy, rather did the patient deteriorate.

Patients with MSMD are at risk of recurrent and disseminated infections with mycobacteria as well as *Salmonella*, but also other intracellular pathogens [3]. This is due to the fact that IFN-γ and its inducible genes, such as TNF, IL-12 or iNOS are central for mounting an efficient host response to mycobacteria residing within the macrophage phagolysosome.^17^ IL-12Rβ1 deficiency has an incomplete penetrance and age of manifestation, severity and outcome are highly variable [3, 5]. In the here presented case, the patient had not suffered any unusual, recurrent or severe infections in the past.

A strength of this case report is that, retrospectively, the established diagnosis can be clearly discriminated from sarcoidosis. We are confident that the patient did neither acquire the infection under the immunosuppressive treatment she initially received, nor that sarcoidosis was triggered by the infection. This case report is limited by its short follow-up period. At the time of writing, the patient is still receiving treatment and thus we cannot provide information on long-term outcome.

In this report we present a rare case of a disseminated infection with *M. genavense* owing to an IL-12Rβ1*-*associated immunodeficiency in an otherwise healthy 31-year-old female who was initially misdiagnosed with sarcoidosis. Likewise, long-term corticosteroid treatment may have resulted in a further impairment of immune control of mycobacterial infection and uncontrolled bacterial amplification. Sarcoid-like reaction does not equal sarcoidosis, and sarcoidosis remains a diagnosis of exclusion [1]. However, diagnosing latent or subclinical infections may be challenging when pathogens are hardly detectable or not expected in an apparently immunocompetent patient. Patients with MSMD may be asymptomatic until adulthood, and MSMD or alternative genetic defects of mycobacterial immune control should be suspected in patients with disseminated mycobacterial infection without pharmacological immunosuppression or acquired immunodeficiency.

## Data Availability

Not applicable.

## Abbreviations

ACE: Angiotensin converting enzyme
^18^FDG-PET/CT: ^18^FDG-positron emission tomography/computed tomography
HIV: Human immunodeficiency virus
IFN-γ: Interferon gamma
IL-12: Interleukin 12
IL-12Rβ1: Interleukin 12 receptor subunit beta 1
iNOS: Inducible nitric oxide synthase
MSMD: Mendelian susceptibility to mycobacterial diseases
PCR: Polymerase chain reaction
sIL2R: Soluble interleukin 2 receptor
TNF: Tumor necrosis factor
